# Transgenerational Sterility of a Transgenic *C. elegans* : A Cautionary Tale

**DOI:** 10.17912/micropub.biology.001343

**Published:** 2024-11-08

**Authors:** Zachary Leydig, Judith Yanowitz

**Affiliations:** 1 Magee-Womens Research Institute, Pittsburgh, Pennsylvania, United States; 2 Dept. of Biology, University of Pittsburgh, Pittsburgh, Pennsylvania, United States; 3 Dept. of Obstetrics, Gynecology and Reproductive Sciences, University of Pittsburgh School of Medicine

## Abstract

The integration of fluorescent protein tags (GFP, mScarlet, mNeon, etc) by CRISPR is often inefficient due to the size of the tags (hundreds of base pairs). To facilitate fluorescent tagging, the split-GFP and split-Scarlet systems have been optimized for use in *C. elegans* (Goudeau et al., 2021). In *C. elegans* , *glh-1 ::T2A::wrmScarlet(1-10)* allows germline gene expression of red fusion proteins. While initial studies reported wild-type broods at both permissive and non-permissive temperatures, we report here that at least one such transgene confers sterility after continuous culturing at 25.5 °C. This serves as a cautionary tale for use of the *glh-1* driver and/or T2A::mScarlet fusions to this protein.

**Figure 1. split-wrmScarlet(1-10) worms exhibit transgenerational sterility:**
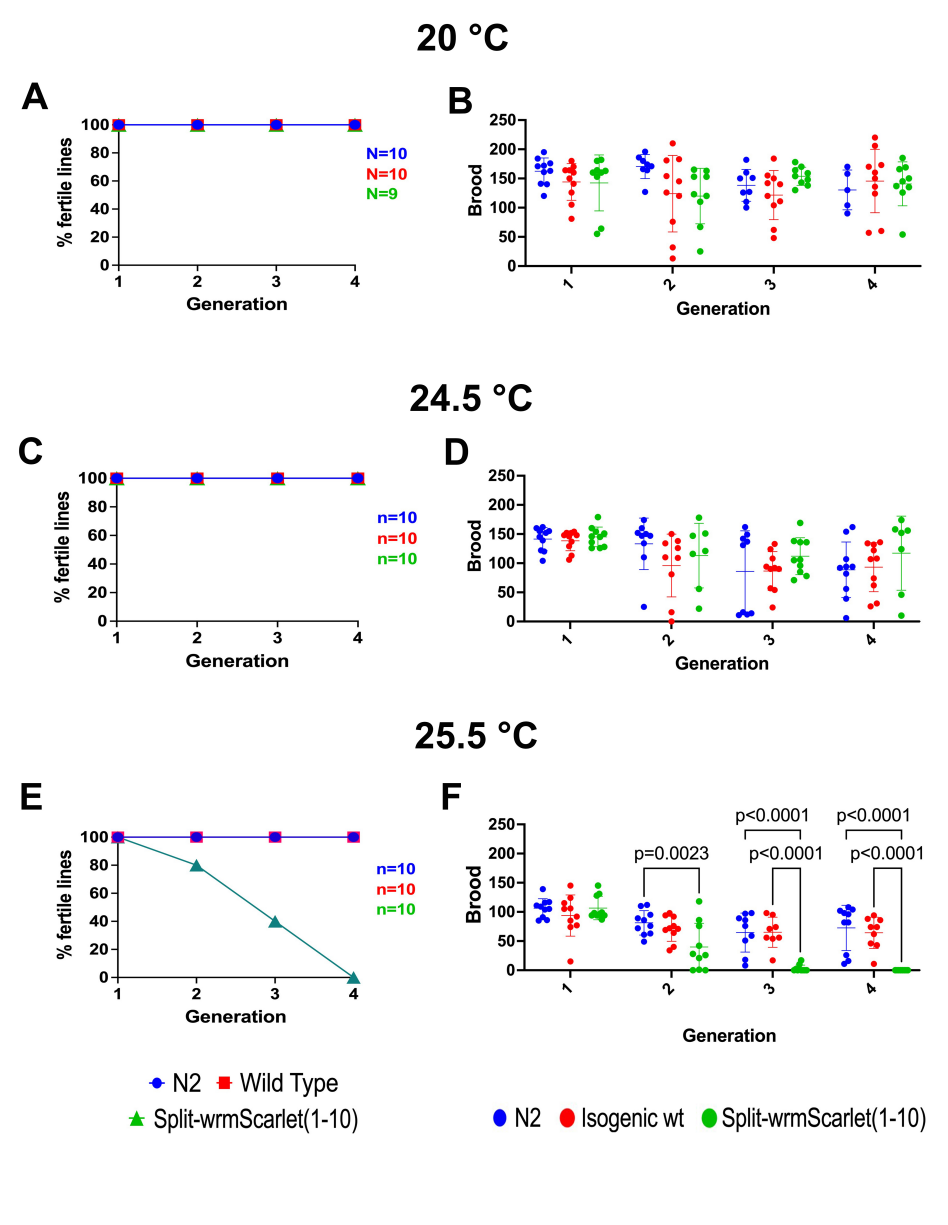
(A-D) *split-wrmScarlet(1-10)* worm populations maintain fertility at 20 °C and 24.5 °C similar to N2 and isogenic wt at 20˚C and 24.5˚C. (E, F) *split-wrmScarlet(1-10)* worm populations show decreased fertility upon passaging at 25.5 °C with complete sterility achieved in *split-wrmScarlet(1-10)* worms by generation 5. N2 and the isogenic wild type populations exhibit sub-fertility, but remain fertile. (B, D, F) Bars indicate mean with standard deviation. Number of lines counted is represented by number of circles color coded to genotype. Statistical significance is shown for intragenerational comparisons between genotypes that are p<.01 (99% CI) using a 2-way ANOVA with Tukey's multiple comparisons test.

## Description

With the explosion of CRISPR genome engineering, creation of endogenously-tagged fluorescent proteins for any gene has become feasible. The split self-associating fluorescent proteins (split-FPs) takes advantage of the ability of the first 10 strands of a beta barrel (FP1-10) to join with the final beta sheet (FP11) to form the functional fluorophore (Cabantous et al., 2005). To facilitate strain construction, both GFP and mScarlet FP1-10 have been integrated into the genome and the small FP11 can be added by CRISPR to the protein of interest.

In *C. elegans ,* the first 10 beta sheets were integrated into the genome fused to *glh-1* , a highly expressed germ-line specific helicase ( *glh-1 ::T2A::wrmScarlet(1-10)* (hereafter referred to as “ *split-wrmScarlet(1-10)* ”) (Goudeau et al., 2021; Marnik et al., 2019) . A self-cleaving peptide from *thosea asigna virus 2A* (Liu et al., 2017) , T2A, bridges GLH-1 and split-wrmScarlet(1-10), allowing the FP1-10 to be released. FP1-10 then finds and binds to FP11 _,_ creating the functional mScarlet fluorophore fused to the protein of interest.

Before making fusion proteins, we wanted to ensure that the *split-wrmScarlet(1-10)* transgenic strain had no reproductive defects at elevated growth temperatures of 24.5 °C and 25.5 °C. Reproductive mutations, including those studied in our laboratory, frequently show more penetrant phenotypes at higher temperatures. Furthermore, since our lab has worked with mortal germ line (Mrt) mutations (e.g. McClendon et al., 2016; Yanowitz, 2008), it was critical for us to test the functionality of the strain across generations. For comparisons, we used both our lab N2 stock and an isogenic wild-type strain from the outcrossed *split-wrmScarlet(1-10)* strain. The latter was used in addition to N2 as a way of - at least partially - eliminating concerns that any phenotypes we observe are due to the *split-wrmScarlet* and not to a background mutation that arose in the strain. At 20 ˚C and 24.5 ˚C, transgenerational analysis showed that the *split-wrmScarlet(1-10)* strain behaved like the N2 and the isogenic wild-type ( *wt* ) strains with no sterility after four generations (Fig 1A-D). We note that the stocks remained fertile after long-term passaging at both temperatures. At 25.5 °C. a slight decrease in brood size is observed in N2 and the isogenic *wt* as expected (Petrella, 2014) . By contrast, almost all of the *split-wrmScarlet(1-10)* lines went sterile by the fourth generation (Fig 1E, F) and the final line had fewer than ten offspring, all of which were slow-growing and sterile. Thus, *split-wrmScarlet(1-10)* exhibits a fully penetrant Mrt phenotype by the F5 generation at 25.5. °C.

Prior studies have shown that RNAi knockdown of *glh-1* causes sterility in F1 offspring at high temperatures (Kuznicki et al., 2000) and that deletion mutations in *glh-1* display ~10% sterility at 20 °C and almost complete sterility in M ^-^ Z ^-^ offspring at 26 °C (Spike et al., 2008) . We hypothesize that the *glh-1 ::T2A::wrmScarlet(1-10)* allele causes a reduction in function of the GLH-1 protein at high temperatures. This could result if the 20 amino acids that remain after cleavage by T2A interferes with GLH-1 function at elevated temperatures. Alternatively, the self-cleaving T2A may not function properly at elevated temperatures, leaving FP1-10 attached to a subset of GLH-1 proteins. Although the similarly crafted split-GFP transgenes was not tested, one might expect a similar phenotypic outcome if the defect is caused by an issue with T2A cleavage. Alternatively, the transgenerational sterility may be due to the *split-wrmScarlet(1-10)* itself. Future experiments comparing the split-GFP and split-Scarlet would help tease out the causative agent for transgenerational sterility. Regardless of mechanism, either possibility results in a loss-of-function (lof) allele at the higher temperature for *split-wrmScarlet(1-10)* . Since complete loss of *glh-1* confers maternal-effect sterility, the *glh-1 (lof)* effects may be more subtle, explaining the loss of fertility beginning in the F3. The transgenerational sterility may only be seen at the higher temperatures since *glh-1* mutations are themselves inherently temperature sensitive.

In summary, we show that there is a strong transgenerational sterility phenotype for the *split-wrmScarlet(1-10)* worms at 25.5 ˚C. This is clearly temperature dependent, as the population maintains fertility at 20 ˚C and 24.5 ˚C. However, we cannot preclude the possibility that *glh-1 ::T2A::wrmScarlet(1-10)* worms would exhibit sterility if passaged singly for >30 generations at the lower temperatures as well. Therefore, we urge caution when tagging proteins with split-wrmScarlet using the *glh-1 ::T2A::wrmScarlet(1-10)* background. This also serves as a critical reminder that functionality of fusion proteins needs to be addressed across the range of viable temperatures and across generations.

## Methods

***C. elegans* husbandry**

Animals were grown on MYOB Peptone plates (for 3L: 51 grams of agar powder (Thermo Scientific cat. No:A10752.0E); 9 grams of bacto-peptone (Gibco cat. No:211677); 6 grams of NaCl (Fisher cat. No:S271-10); 1.65 grams of Tris HCl (JT Baker manufacture part No: Jt-4103-01); 0.72 Trizma Base (Sigma CAS No: 77-86-1)). Cholesterol was added after autoclaving, just prior to plating. The *E. coli* OP50 strain was used as a food source. Plates were kept at 20 ˚C on 6 cm plates prior to temperature shift experiments.

**Strains Utilized**

Strains utilized in this study were N2 , DUP237 : *glh-1 ( sam140 [ glh-1 ::T2A::wrmScarlet(1-10)]* , QP2537: two times outcrossed DUP237- *glh-1 ( sam140 [ glh-1 ::T2A::wrmScarlet(1-10)]* , and QP2540: isogenic wild type (wt) obtained from outcrossing DUP237 to N2 . N2 and DUP237 were obtained from the *Caenorhabditis* Genetic Center (CGC). QP2537 is referred to as *split-wrmScarlet(1-10)* herein. Genotypes were confirmed use the following PCR primers.

Flanking split-wrmScarlet(1-10)

(Fwd: 5' AGCATTGTTTTCAGAACTGGAAGAGTTGGA 3')

(Rev: 5' CAGCATGATATTGAACAGAGTTTATGCGGC 3')

Forward primer within 1-10 used with flanking reverse primer

(Fwd: 5' CCCACCAGATGGACCAGTTATG 3' )

An isogenic wild-type strain from the outcrosses was used as a control (referred to as isogenic wild type (wt)) alongside N2 to rule out background effects in the original strain. Although the results presented herein represent data from N = 10 of the outcrossed lines, we initially observed the sterility multiple times in the DUP237 line obtained directly from the CGC, motivating the outcrossing and more careful analysis.

**Brood size assays**

Ten N2 , isogenic wild type, or *split-wrmScarlet(1-10)* L4 hermaphrodites were individually plated on 3 cm MyoB plates center seeded with OP50 . Four days later the F1 offspring (L2 or beyond) were counted. To assess brood size changes across generations, at day three (24.5˚C and 25.5˚C) or day four (20.0˚C), one L4 hermaphrodite from each plate was transferred to a new 3 cm plate. If no offspring are seen and the transferred worm could not be found (e.g. it crawled up the side of the plate and desiccated), siblings from the prior generation were plated to maintain the population. If no fertile sister worms could be recovered, the stock was marked as sterile. If no sister worms could be recovered because the plate had starved, the line was marked as fertile.

**Thermocoupler temperature checks**

The HH506RA Multilogger thermometer was used to check the true plate temperature during random plate checks and passaging/scoring of the worms. This was done by physically sticking the probe into the agar of a test plate on the same rack immediately after taking the plate out of the incubator. Temperature checks were performed for all temperatures and recorded (Table 1).

**Table 1. Natural variation in incubator temperatures.**

**Table:** 

20 °C incubator	24.5 °C incubator	25.5 °C incubator
21.2	24.2	25.5
21.4	24.2	25.6
19.8	24	25.4
21.3	24	25.5
21.8	24.6	25.6
21.7	24.8	25.4
20.6	24.8	25.5
21.4	24.3	25.5
21.4	24.6	25.6
